# Factors influencing the use of modern contraceptive in Nigeria: a multilevel logistic analysis using linked data from performance monitoring and accountability 2020

**DOI:** 10.1186/s12905-020-01059-6

**Published:** 2020-09-03

**Authors:** Oluwafemi David Alo, Babajide Oluseyi Daini, Olugbenga K. Omisile, Ebere Joy Ubah, Odunayo Esther Adelusi, Ochanya Idoko-Asuelimhen

**Affiliations:** 1 Marie Stopes International Organisation Nigeria, Abuja, Nigeria; 2 Population Council, Abuja, Nigeria; 3 Africa Leadership Forum, Abuja, Nigeria

**Keywords:** Contraceptives, Family planning, Multilevel, Nigeria

## Abstract

**Background:**

The population of Nigeria is estimated at over 190 million and it is projected to increase by 44% between 2015 and 2030. However, less than one-quarter of women within reproductive age in Nigeria uses modern contraceptive methods despite its importance. Hence, this study aims at examining the influence of individual and community level factors on the use of modern contraceptive method.

**Methods:**

The study is a secondary analysis of linked household and Service Delivery Point datasets from a 2018 survey conducted by Performance, Monitoring and Accountability in Nigeria. Data was abstracted for a total of 9126 sexually active women within the ages of 15–49 years across 295 enumeration areas in seven States. A 2-level binary logistic regression was used to examine the association between study variables and the use of modern contraceptives while adjusting for the clustering effect.

**Results:**

There was significant influence of educational level, marital status, parity, socio-economic status, fertility intention, and awareness of family planning methods on the use of modern contraceptives. Also, women who perceived support from someone in the community on family planning were more likely to use modern contraceptive unlike those without such support. Those who believed that contraceptive methods are used by almost all and some of their friends or relatives were more likely to use modern contraceptive compared to those who think otherwise.

**Conclusions:**

The study shows the need to reduce inequalities between FP utilization across women with different socio-economic status as well as increasing the awareness for modern contraceptive methods.

## Background

In 2015, the population of the world was estimated at 7.3 billion and projected to increase to 8.5 billion by 2030. This projection’s degree of uncertainty depends majorly on future levels of fertility in countries with high-fertility including Nigeria which as at 2016 had a total fertility rate of 5.53 children per woman. The population of Nigeria is estimated at over 190 million and it is projected to increase by 44% between 2015 and 2030 [1].

The use of contraceptives is essential in slowing unsustainable population growth and reduction of maternal morbidity and mortality by preventing ill-timed pregnancies and births [2, 3]. However, past studies have shown a low uptake of contraceptive methods and high unmet needs in Nigeria [4–7]. The most recent National Demographic and Household survey reported a 17% contraceptive prevalence rate among married women between ages 15 to 49 years with only 12% users of modern contraceptive methods. Although, the use of contraceptive methods was higher (37%) among sexually active unmarried women with 28% using a modern method [8].

Previous studies have shown evidence of the influence of age, number of children, educational level, socio-economic status, fertility intention, cultural belief, awareness of Family Planning (FP) methods, fear of side effects, partner’s disapproval, misconceptions and myths on low uptake of modern contraceptive methods [9–13]. However, no study has examined the relationship between the use of modern contraceptive and women’s perception of being favoured by someone in the community to use FP, awareness of contraceptive use among friends or family, recent visitation by community health worker on FP and attendance in community gathering where FP was favoured. Based on this background, this study aims at examining how the use of modern contraceptive method is influenced by socio-demographic characteristics, awareness of FP methods, women perception on being favoured to use FP method by someone in the community, attendance in community event where FP was favoured, awareness of contraceptive use among friends and family, hearing community leaders or spiritual leaders talk in favour of FP, availability of health facility that provide free FP services in the community, average number of days FP supported facilities in the community are opened and number of health facilities providing FP services in the community (access).

## Methods

### Study design

The study is a secondary analysis of linked household and Service Delivery Point (SDP) datasets from a survey conducted by Performance, Monitoring and Accountability (PMA2020) in Nigeria.

### Data

Data were extracted from round 5 of Nigeria’s PMA2020 survey conducted in 2018. A two-stage cluster design was used within a sample of seven states (Kaduna, Kano, Lagos, Rivers, Nasarawa, Taraba, and Anambra). One state was selected in each of the six geopolitical zones while probability proportional to size was used to select the seventh state (Kaduna). Thirty-five to forty (35–40) households were randomly selected from 302 enumeration areas (EAs) which were drawn from the National Population Commission’s master sampling frame. Data was collected between April and May 2018 from eligible females of reproductive age (15–49) in the household who consented for an interview. Similarly, data was collected from health facilities that fall within the boundaries of the EA. Details on data collection methods, instruments, ethical, data quality assurance are provided elsewhere [14]. Data from the household and SDP surveys were merged using the cluster-ID variable in the two datasets. Data was merged and abstracted for a total of 9126 sexually active women within reproductive age in 295 EAs.

### Study variables

The dependent variable is use of modern contraceptive methods in the last 12 months, categorized as ‘Yes’ for IUD, implants, injectables, Pills, condoms, diaphragm, cycle beads, and female sterilization users while ‘No’ for those who uses withdrawal, lactational amenorrhea method (LAM), rhythm, and none users of FP methods. Individual-level variables include respondent age, categorized as below 20, 20–29, 30–39 and at least 40 years, marital status classified as currently married, divorced or widow and singles which includes women who are not married but living with their partner. Other variables include respondent’s level of education (below secondary, secondary and tertiary), parity grouped as 0, 1–2, 3–4, 5+, wealth index categorized as below middle class (lowest and lower quintile), middle class, above middle class (higher and highest quintile), awareness of modern FP methods classified as heard about < 5 methods or at least 5 methods and interest in having more children in the future.

Other variables examined in the study are; respondents’ awareness on how many of their friends or relatives uses contraceptives (none or don’t know, some, and most of all), attendance in any community event where FP was favoured, visit to a health facility within 12 months, visitation by health worker on FP, perception on being favoured by someone in the community to use FP methods, heard community leader speaks in favour of FP methods, type of residence, and number of FP supported facilities in the community (at most one, 2, 3+), average number of days in a week FP services are provided in health facilities (at most 3, 4 to 5 days, at least 6 days) and availability of health services that provide free FP services in the community.

### Data analysis

Descriptive statistics were used to summarize study variables. Bivariate analysis was conducted to initially explore the association between study variables and the use of modern contraceptive methods. Due to the sampling method used, the dependence of responses from different levels of hierarchy was suspected which implies a single-level statistical model might not be adequate to control for the clustering effect [15].

Therefore, a two-level binary logistic regression using the individual respondent as level one and community (cluster) as level two with no covariates was used to verify if the magnitude of random effects at the community level justified the use of multilevel regression for the analysis. A decision was based on the Intra-Class Correlation (ICC), which shows the amount of dependency that was observable due to the clustering of data at the community level.

Furthermore, a two-level univariate binary logistic regression was used to examine the unadjusted association between the use of modern contraceptive methods and study variables. Variables with significant association at 10% level of significance were included in a 2-level multivariate logistic regression to examine the adjusted association. Survey weights were adjusted for in order to control for disproportionate sampling and non-responses. Hence, variances were calculated to account for clustering and design effects using Taylor linearization methods. The measure of associations of the individual and community-level factors on the use of modern contraceptive methods were examined using the Odds Ratio (OR) at 95% confidence interval (CI) and the random effects were expressed in variance. We used Stata 15 (Stata Corps) for all analyses.

## Result

A total of 9126 sexually active women aged 15–49 across 295 clusters (community) were included in the study. Modern contraceptives were used by 1755 (19.2%) women of which 27% were condom users, 21% pills, 23% injectables, 22% implants, 4% IUD, 1% female sterilization and about 1% diaphragm and beads. As shown in Table 1, modern contraceptives were 17.1% among currently married women, 12.8% divorced/widow and 28.9% sexually active unmarried women including those who are living with their partners. About one fifth (21.1%) of the modern contraceptive users were between ages of 30–39 years and 13.2% among women below 20 years (Table 1).

**Table 1:** Distribution and association between study variables on utilization of modern contraceptives

Variable	Use of modern FP (%)	Traditional or non-FP users	*P*-Value
Age group			< 0.001
15–19 years	118 (13.2)	776 (86.8)	
20–29 years	685 (19.8)	2768 (80.2)	
30–39 years	632 (21.1)	2361 (78.9)	
40–49 years	320 (17.9)	1466 (82.1)	
Educational level			< 0.001
Below secondary	431 (10.7)	3587 (89.3)	
Secondary	869 (24.6)	2663 (75.4)	
Higher	455 (28.9)	1121 (71.1)	
Marital status			< 0.001
Single/living with partner	523 (28.9)	1287 (71.1)	
Currently married	1165 (17.1)	5639 (82.9)	
Divorced/Widow/Separated	65 (12.8)	445 (87.2)	
Parity			< 0.001
None	398 (20.5)	1542 (79.5)	
1 to 2	410 (15.7)	2195 (84.3)	
3 to 4	504 (21.8)	1807 (78.2)	
5 and above	440 (19.5)	1814 (80.5)	
Interest in more children			< 0.001
More children	1020 (17.2)	4901 (82.8)	
Maybe/No	734 (23.1)	2448 (76.9)	
Household wealth quintile			< 0.001
Below average quintile	707 (14.2)	4258 (85.8)	
Average quintile	339 (22.8)	1146 (77.2)	
Above average quintile	709 (26.5)	1967 (73.5)	
Number of modern FP methods heard of			< 0.001
at most 4	231 (7.9)	2695 (92.1)	
at least 5	1524 (24.6)	4676 (75.4)	
Visited by health worker about FP in last 12 months			< 0.001
Yes	331 (26.5)	919 (73.5)	
No	1423 (18.2)	6418 (81.8)	
Facility visit in the last 12 months			< 0.001
No visit	625 (15.9)	3315 (84.1)	
Visited but No FP talk	472 (17.6)	2206 (82.4)	
Visited and FP talk	658 (26.4)	1832 (73.6)	
Attended a community event where FP were favoured			< 0.001
Yes	328 (28.9)	806 (71.1)	
No	1426 (17.9)	6533 (82.1)	
Someone in the community favour you if you use FP			< 0.001
Yes	926 (30.3)	2130 (69.7)	
No	827 (13.7)	5215 (86.3)	
Heard community/religious leader speak in favour of FP			< 0.001
Yes	868 (25.0)	2607 (75.0)	
No	874 (15.7)	4685 (84.3)	
How many of your close friends or family do you think uses FP			< 0.001
None/Unknown	381 (9.0)	3847 (91.0)	
Some	983 (25.6)	2855 (74.4)	
Most/all	391 (37.7)	647 (62.2)	
Type of residence			< 0.001
Urban	998 (24.1)	3146 (75.9)	
Rural	757 (15.2)	4225 (84.4)	
No of facility offering FP services in the community			< 0.001
at most 1	347 (17.4)	1649 (82.6)	
two	532 (18.6)	2327 (81.4)	
at least 3	876 (20.5)	3395 (79.5)	
Average number days FP facilities in the communities are opened			< 0.001
At most 3 days	167 (16.1)	869 (83.9)	
4 to 5 days	703 (18.2)	3166 (81.8)	
at least 6 days	885 (21.0)	3336 (79.0)	
Presence of at least one facility that provide Free FP services			< 0.001
Yes	1356 (20.6)	5234 (79.4)	
No	399 (15.7)	2137 (84.3)	

The univariate analysis showed a significant association (*p* < 0.1) of women’s age, educational level, marital status, parity, fertility preference, household wealth quintile, health worker visitation on FP, facility visit and attendance in FP community event on the use of modern contraceptives. Also, being favoured by someone in the community to use FP, hearing community leader speak in favour of FP, awareness of FP usage among friends and relatives, type of residence, number of supported FP facilities, average number of days in a week health facility provide FP services, and presence of health facilities with free FP services were significantly (*p* < 0.1) related to the use of modern contraceptives (Table 1). These variables were subsequently included in the multivariate multilevel model.

The intra-class correlation in the null 2-level binary logistic regression indicated that 20% of the total variance in the use modern contraceptive was attributable to the dependency of observation within the communities, which implies a significant correlation (ICC: 20, 95% C.I: 17–27%) within the communities (Table 2). The 2-level multivariate binary logistic regression in Table 3 showed that women with higher levels of education [aOR: 1.7, 95% C.I: 1.07–2.58] were more likely to use modern contraceptive compared to those who had below secondary level of education.

**Table 2:** Null model for usage of modern FP

Modern FP	OR	Standard error	95% C.I
Constant	0.2	0.02	0.19–0.27^α^
Random effect			
Community level variance	0.9	0.15	0.66–1.24
ICC	0.2	0.03	0.17–0.27^α^

*OR* Odd Ratio, *C.I* Confidence Interval, *ICC* Intra-class correlation

^α^ significant at *p* < 0.05

**Table 3:** Effect of study variables on use of modern FP using two-level binary logistic regression

Variable	Unadjusted estimates		Adjusted estimates	
	Odd ratio	95% C.I	Odd ratio	95% C.I
Age group				
Below 19 years (ref)	1.0		1.0	
20–29 years	1.7	1.09–2.78^α^	1.2	0.62–2.23
30–39 years	1.6	1.00–2.71^α^	0.9	0.41–2.19
40 and above	1.6	0.89–2.71	0.8	0.27–2.49
Educational level				
Below secondary (ref)	1.0		1.0	
Secondary	1.8	1.32–2.48^α^	1.3	0.93–2.01
Higher	2.5	1.84–3.38^α^	1.7	1.07–2.58^β^
Marital status				
Single/living with partner (ref)	1.0		1.0	
Currently married	0.7	0.52–0.89^α^	0.3	0.22–0.45^β^
Divorced/Widow/Separated	0.3	0.16–0.68^α^	0.2	0.08–0.48^β^
Parity				
None (ref)	1.0		1.0	
1 to 2	0.9	0.63–1.34	1.3	0.86–1.97
3 to 4	1.3	1.02–1.76^α^	1.9	1.32–2.80^β^
5 and above	1.4	1.02–1.94^α^	2.3	1.41–3.70^β^
Interest in more children				
More children (ref)	1.0		1.0	
Maybe/No	1.3	1.02–1.70^α^	1.5	1.12–2.01^β^
Household wealth quintile				
Below average quintile (ref)	1.0		1.0	
Average quintile	1.5	1.03–2.17^α^	1.0	0.78–1.54
Above average quintile	2.4	1.88–2.94^α^	1.5	1.06–1.99^β^
Number of modern FP methods heard of				
at most 4 (ref)	1.0		1.0	
at least 5	3.2	2.24–4.46^α^	2.2	1.37–3.39^β^
Visited by health worker about FP in last 12 months				
No (ref)	1.0		1.0	
Yes	1.4	1.12–1.68^α^	0.9	0.68–1.33
Facility visit in the last 12 months				
No visit (ref)	1.0		1.0	
Visited but No FP talk	1.0	0.76–1.26	0.9	0.68–1.26
Visited and FP talk	1.5	1.03–2.21^α^	1.1	0.75–1.74
Attended a community event where FP were favoured				
No (ref)	1.0		1.0	
Yes	1.2	0.83–1.74	0.	0.49–1.23
Someone in the community favour you if you use FP				
No (ref)	1.0		1.0	
Yes	2.8	2.32 - 4.216^α^	2.0	1.59–2.55^β^
Heard community/religious leader speak in favour of FP				
No (ref)	1.0		1.0	
Yes	1.5	1.15–1.82^α^	1.0	0.75–1.32
How many of your close friends or family do you think uses FP				
None/Unknown (ref)	1.0		1.0	
Some	3.5	2.80–4.42^α^	2.7	2.06–3.65^β^
Most/all	5.1	3.49–7.37^α^	3.5	2.25–5.43^β^
Type of residence				
Urban (ref)	1.0		1.0	
Rural	0.5	0.34–0.65^α^	1.1	0.69–1.62
No of facility offering FP services in the community				
at most 1 (ref)	1.0		1.0	
two	1.7	1.09–2.65^α^	1.2	0.84–1.90
at least 3	1.4	0.89–2.11	1.0	0.65–1.50
Average number days FP facilities in the communities are opened				
At most 3 days (ref)	1.0		1.0	
4 to 5 days	2.1	1.16–3.91^α^	1.1	0.59–2.29
at least 6 days	2.1	1.13–3.78^α^	1.0	0.50–1.96
Presence of at least one facility that provide Free FP services				
No (ref)	1.0		1.0	
Yes	1.6	1.15–2.12^α^	1.1	0.80–1.62
Random effect				
Variance	–	–	0.5	0.36–0.78^β^

*OR* Odd Ratio, *ref.* reference category, *C.I* Confidence Interval

^α^ significant at *p* < 0.1, ^β^significant at *p* < 0.05

Also, women who are currently married [aOR: 0.3, 95% C.I: 0.22–0.45] and those who are divorced or widow [aOR: 0.2, 95% C.I: 0.08–0.48] were less likely to use modern contraceptives unlike sexually active unmarried women. The likelihood of using modern contraceptives increases with parity level; women with parity between 1 and 2 [aOR: 1.3, 95% C.I: 0.86–1.97], 3–4 [aOR: 1.9, 95% C.I: 1.32–2.80] and at least 5 [aOR: 2.3, 95% C.I: 1.41–3.70] are more likely to use modern contraceptives compared to women with zero parity.

Women who have no intention of having more children [aOR: 1.5, 95% C.I: 1.12–2.01] were more likely to use modern contraceptive compared to those who desire more children. There was no significant difference in the likelihood of using modern contraceptive between those who are in the average wealth quintile [aOR: 1.0, 95% C.I: 0.78–1.54] and those below the average quintile. However, women above the average quintile [aOR: 1.5, 95% C.I: 1.06–1.99] were more likely to use modern contraceptive compared to those below the average quintile.

Women who are aware of more types of FP methods [aOR: 2.2, 95% C.I: 1.37–3.39] were more like to use modern contraceptive compared to women who have heard about fewer types of FP methods. Also, women who perceived support from someone in the community regarding FP [aOR: 2.0, 95% C.I: 1.59–2.55] were more likely to use modern contraceptive unlike those without such support (Table 3).

Furthermore, women who believed that contraceptive methods are used by most [aOR: 3.5, 95% C.I: 2.25–5.43] and some [aOR: 2.7, 95% C.I: 2.06–3.65] of their friends and relatives were more likely to use modern contraceptive compared to those who think otherwise. However, there was no evidence of statistically significant [*p* > 0.05] influence of visitation by health worker concerning FP, facility visit with or without FP talk, attendance in a community event where FP was favoured, hearing community leader speaking in favour of FP, type of residence, average number of days FP supported facilities are opened, presence of facility that provide free FP services in the community and number of FP supported facilities on the use of modern contraceptive (Table 3).

## Discussion

Findings from this study shows that uptake of modern contraceptive method has slightly improved (19%) from previous years in Nigeria: 10% in 2012 [6], 11% in 2013 [5]. Improvements have shown the commitment and investments made in achieving Nigeria FP2020 commitment of 27% rate of modern contraceptive use [8].

Our study shows that age was not a significant factor influencing FP use among sexually active women. This was in contrast with findings from other studies [16, 17]. The likelihood of modern FP use was lower among widow or divorced and currently married women compared to those who are unmarried but sexually active. Possible explanations for this might be lack of partner support on FP use [18, 19] lack of married women independency in making beneficial reproductive health decisions [20] and the need to avoid stigmatisation as a result of unwanted pregnancies in a non-marital relationship [21, 22].

Inequality in family planning service uptake has been reported as far back as the early 2000’s. A multi-country study conducted by Health policy initiative in 2007 across 47 developing countries revealed the inequalities in the use of family planning, unmet need for family planning, use of maternal health services, social trends and birth spacing in these countries [23]. These inequalities have remained the same over the years despite numerous interventions and investments aimed at targeting the poor and less privileged who are characterized as someone with lower educational levels and living in rural or hard to reach areas [24, 25]. Our findings also show similar patterns with these studies, with those who are in the higher economic level and those above secondary education being more likely to use modern family planning compared to the lesser groups. Better health-seeking behaviour among the more enlightened and privileged might be another possible explanation [26].

Also, the likelihood of using modern FP increases from women who had 3–4 children to at least 5 children compared to women who are yet to have a child. Similarly, women with fertility intentions are less likely to use modern contraceptives as child spacing and prevention of unwanted pregnancy is the main reason for contraceptive use. The use of modern FP methods was significantly higher among women who are aware of more FP methods. This was similar to findings from other study [16].

The study also shows a significant influence of support from community member on the use of modern FP. Such support might impact positively on the woman’s perception of FP and willingness to uptake any modern FP methods. Also, uptake of modern FP was more likely among women who perceived that most or all their relatives and friends are using any FP methods. Such perception could help the women overcome any fear of side effects, cultural bias and increase their confidence on the effectiveness of FP.

However, there was no significant influence of visitation by health worker on FP and health facility visit on the use of modern FP. This might be due to negative experiences or interactions with health care providers which could affect how the women process FP information provided by health care worker [27, 28]. Similarly, attending a community event where FP was favoured and hearing community or religious leader speak in favour of FP did not significantly influence the use of modern FP. Although, there was no available information to access the focus or agenda of such community events or the level of information on FP that was shared by community or religious leader. Community events without clear FP agenda and sharing of inadequate information on FP by community or religious leader might be insufficient in improving the uptake of modern FP.

Furthermore, there was no significant association between the use of modern FP and type of residence, number of facilities offering FP services, average number of days in a week FP facility are functional, and presence of at least a facility that provides free FP services.

## Conclusion

Uptake of modern contraceptive methods is still below Nigeria’s commitment of 27% mCPR in 2020. In order to achieve this, targeted interventions should be implemented to increase the uptake of modern contraceptives among sexually active women (especially married women). It is also important to adopt strategies that combine individual education, improvement of services and community outreach/mobilization to inform communities about available services, need for child spacing and to increase acceptability and use of family planning. This may also include targeting male influence and attempt to sway it in favour of family planning while improving agency of married women to make positive reproductive health choices.

Also, the study shows the need to reduce inequalities between FP utilization across women with different socio-economic status. Sustainable programs including FP focused outreach with provision of affordable FP services should be implemented especially in hard to reach communities. Furthermore, community FP focused support group should be encouraged because women seem to be influenced by support to use FP methods.

The data used for this study may have been subjected to a few biases, including recall bias and social desirability bias during the data collection process. There was also no detail information about the FP supported events in the community, type of FP information shared by community or religious leader, respondent perception and experiences with health care provider and other community level factors in the dataset. Despite the study findings, there is need for a mixed-method research to explore the role of community level factors on the uptake of modern contraceptives in Nigeria.

## Data Availability

The data sets and corresponding survey instruments used in this study are free to download upon approved request through the PMA2020 website https://www.pma2020.org/request-access-to-datasets

## Abbreviations

FP: Family Planning
SDP: Service Delivery Point
PMA: Performance, Monitoring and Accountability
EA: Enumeration Area
IUD: Intrauterine Device
LAM: Lactational Amenorrhea Method
ICC: Intra-Class Correlation
aOR: Adjusted Odd Ratio
CI: Confidence Interval
CPR: Modern Contraceptive Prevalence Rate

## References

[1] Hertog S, Cohen B. Population 2030. Demographic challenges and opportunities for sustainable development planning: United Nations; 2015. https://www.un.org/en/development/desa/population/publications/pdf/trends/Population2030.pdf.

[2] Kavanaugh ML, Anderson RM (2013). Contraception and beyond: the health benefits of services provided at family planning centers.

[3] WHO (2015). Family planning /contraception, media centre fact sheets.

[4] Asekun-Olarinmoye E, Adebimpe W, Bamidele J, Odu O, Asekun-Olarinmoye I, Ojofeitimi E (2013). Barriers to use of modern contraceptives among women in an inner-city area of Osogbo metropolis, Osun state, Nigeria. Int J Women's Health.

[5] National Population Commission (NPC), ICF International (2014). Nigeria demographic and health survey 2013.

[6] Federal Ministry of Health [Nigeria] (2013). National HIV/AIDS and reproductive health survey, 2012 (NARHS Plus).

[7] Babalola S, Oyenubi O, Speizer IS (2017). Factors affecting the achievement of fertility intentions in urban Nigeria: analysis of longitudinal data. BMC Public Health.

[8] National Population Commission – NPC/Nigeria, ICF (2019). Nigeria demographic and health survey 2018.

[9] Ibisomi L (2014). Is age difference between partners associated with contraceptive use among married couples in Nigeria?. Int Perspect Sex Reprod Health.

[10] Adebowale SA, Adedini SA, Ibisomi LD, Palamuleni ME (2014). Differential effect of wealth quintile on modern contraceptive use and fertility: evidence from Malawian women. BMC Womens Health.

[11] Okigbo C, Speizer I, Domino M, Curtis S (2017). A multilevel logit estimation of factors associated with modern contraception in urban Nigeria. World Med Health Policy.

[12] Lakew Y, Reda AA, Tamene H, Benedict S, Deribe K (2013). Geographical variation and factors influencing modern contraceptive use among married women in Ethiopia: evidence from a national population-based survey. Reprod Health.

[13] Hindin MJ, McGough LJ, Adanu RM (2014). Misperceptions, misinformation and myths about modern contraceptive use in Ghana. J Family Plann Reprod Health Care.

[14] Zimmerman L, Olson H, Tsui A, Radloff S, PMA2020 Principal Investigators Group (2017). PMA2020: rapid turn-around survey data to monitor family planning service and practice in ten countries. Stud Fam Plan.

[15] Khan MHR, Shaw JEH (2011). Multilevel logistic regression analysis applied to binary contraceptive prevalence data. J Data Sci.

[16] Semachew KA, Tarekegn M, Embiale N (2018). Knowledge, attitude and practice towards family planning among reproductive age women in a resource limited setting of Northwest Ethiopia. BMC Res Notes.

[17] Essiben F, Meka EN, Foumane P, et al. Factors preventing the use of modern contraceptive methods in sexually active adolescents in Yaounde. Obstet Gynaecol Rep. 2018;2(1):1–5. 10.15761/OGR.1000121..

[18] Olaitan OL (2011). Factors influencing the choice of family planning among couples in Southwest Nigeria. Int J Med Med Sci.

[19] Ouma S, Turyasima M, Acca H (2015). Obstacles to family planning use among rural women in atiak health center iv, Amuru district, northern Uganda. East Afr Med J.

[20] Ewerling F, Lynch JW, Victora CG, van Eerdewijk A, Tyszler M, Barros AJ (2017). The SWPER index for women’s empowerment in Africa: development and validation of an index based on survey data. Lancet Glob Health.

[21] Ilika A, Anthony I (2004). Unintended pregnancy among unmarried adolescents and young women in Anambra State, South East Nigeria. Afr J Reprod Health.

[22] Fubam RM, Odukogbe AA, Dairo MD (2019). Psychological and social effects of pregnancy in unmarried young women in Bui, Northwest, Cameroon. Am J Biomed Life Sci.

[23] United States Agency for International Development (2005). Inequalities in the use of family planning and reproductive health services: implications for policies and programs.

[24] Ogundele OJ, Pavlova M, Groot W (2018). Examining trends in inequality in the use of reproductive health care services in Ghana and Nigeria. BMC Pregnancy Childbirth.

[25] Yigzaw M, Zakus D, Tadesse Y (2015). Paving the way for universal family planning coverage in Ethiopia: an analysis of wealth related inequality. Int J Equity Health.

[26] Latunji OO, Akinyemi OO (2018). Factors influencing health-seeking behaviour among civil servants in Ibadan, Nigeria. Ann Ibadan Postgrad Med.

[27] Chi PC, Bulage P, Urdal H, Sundby J (2015). A qualitative study exploring the determinants of maternal health service uptake in post-conflict Burundi and Northern Uganda. BMC Pregnancy Childbirth.

[28] Kiura AW (2014). Constrained agency on contraceptive use among Somali refugee women in the Kakuma refugee camp in Kenya gender. Technol Dev.

